# Human-Inspired Gait and Jumping Motion Generation for Bipedal Robots Using Model Predictive Control

**DOI:** 10.3390/biomimetics10010017

**Published:** 2025-01-01

**Authors:** Zhen Xu, Jianan Xie, Kenji Hashimoto

**Affiliations:** Graduate School of Information, Production and Systems, Waseda University, 2-7 Hibikino, Wakamatsu-ku, Kitakyushu 808-0135, Japan; davidxu@asagi.waseda.jp (Z.X.); xiejn@akane.waseda.jp (J.X.)

**Keywords:** bipedal robots, model predictive control, shooting method, dynamic constraints

## Abstract

In recent years, humanoid robot technology has been developing rapidly due to the need for robots to collaborate with humans or replace them in various tasks, requiring them to operate in complex human environments and placing high demands on their mobility. Developing humanoid robots with human-like walking and hopping abilities has become a key research focus, as these capabilities enable robots to move and perform tasks more efficiently in diverse and unpredictable environments, with significant applications in daily life, industrial operations, and disaster rescue. Currently, methods based on hybrid zero dynamics and reinforcement learning have been employed to enhance the walking and hopping capabilities of humanoid robots; however, model predictive control (MPC) presents two significant advantages: it can adapt to more complex task requirements and environmental conditions, and it allows for various walking and hopping patterns without extensive training and redesign. The objective of this study is to develop a bipedal robot controller using shooting method-based MPC to achieve human-like walking and hopping abilities, aiming to address the limitations of the existing methods and provide a new approach to enhancing robot mobility.

## 1. Introduction

### 1.1. Background

**Figure 1:**
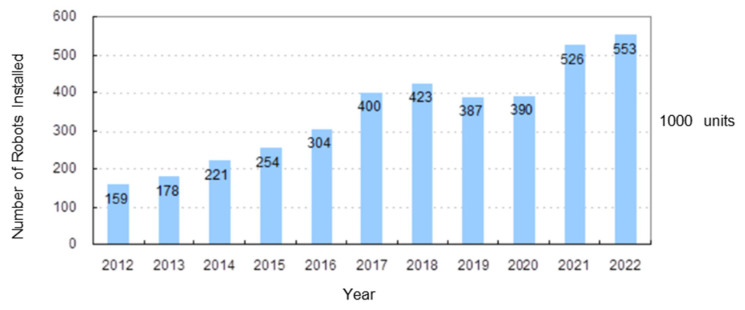
Annual installations of industrial robots from 2012 to 2022.

Since 2017, the robotics industry has experienced rapid growth, with an increasing deployment of robots in practical applications. According to the International Federation of Robotics (IFR) World Robotics 2023 Report, the global sales of industrial robots reached 553,052 units in 2022, representing a 5% increase compared with 2021 and setting a new record (Figure 1). From 2017 to 2022, the compound annual growth rate (CAGR) of global industrial robot sales was approximately 7% [1]. However, it is worth noting that the global COVID-19 pandemic during this period significantly disrupted global supply chains and industrial production. Despite these challenges, the robotics industry demonstrated resilience, with a notable recovery in demand post-2021 as industries accelerated automation efforts to address labor shortages and enhance operational efficiency. These trends highlight the field’s significant potential and the swift integration of robotics technology across various industries, driving global industrial development. The extensive use of industrial robots has improved manufacturing efficiency, enhanced product quality, reduced costs, and fostered the advancement of smart manufacturing and automation, thus creating new avenues for economic growth. These indicators suggest that the robotics industry will continue to expand rapidly, driven by ongoing technological innovation and the broadening of application boundaries.

The recent advancements in robotics have been substantial across multiple domains, leading to an increasingly pivotal role in daily life [2]. While wheeled robots currently dominate due to their stability, controllability, efficiency, and cost-effectiveness, which make them ideal for sectors that require high performance and reliability, legged robots—particularly humanoid robots—offer broader and more profound application potential [3]. However, it is important to recognize that the distinction between wheeled and legged robots is not strictly binary. Instead, there exists a spectrum of designs that combine features from both. For instance, wheeled robots with advanced suspension systems and hybrid mobility mechanisms can outperform legged robots in specific tasks. A notable example is navigating stairs at high speeds: wheeled robots can descend stairs more efficiently and stably by maintaining continuous ground contact and leveraging their mechanical simplicity, whereas legged robots often require complex coordination and careful balance adjustments, which can slow down their movement. By understanding this spectrum and analyzing task-specific advantages, the application potential of these designs can be better aligned with real-world requirements.

Humanoid robots exhibit notable adaptability and flexibility, which, in principle, enable them to navigate crowded areas and react to obstacles and hazards using advanced sensors and algorithms [4]. These characteristics could make them suitable for environments requiring close human interaction, such as in the service industry, healthcare, and education. However, it is important to acknowledge that achieving human-level adaptability and navigation capabilities remains a significant challenge. Current humanoid robots often face difficulties in real-world scenarios, such as maintaining balance on uneven surfaces or reacting promptly to unexpected changes in dynamic environments.

Their potential advantages are particularly evident in hazardous or extreme environments, where their ability to access difficult-to-reach areas may reduce risks to human safety. For example, humanoid robots have been proposed for disaster rescue operations, where they could navigate rubble to locate survivors and perform complex tasks, or in nuclear plant inspections, where they could minimize human exposure to radiation by conducting equipment checks and repairs. However, these applications are still largely experimental, and their practical implementation depends on further advancements in reliability and autonomy.

In summary, while the development of humanoid robots holds promise, particularly in optimizing motion planning, it is critical to address the current limitations to improve their operational effectiveness across various scenarios. Enhancing motion planning and navigation capabilities will not only increase their practicality and reliability but also expand their range of applications, ultimately providing greater safety and convenience to society.

### 1.2. Existing Control Methods for Bipedal Robot Gait Generation

Currently, methods for generating bipedal robot gaits have achieved significant success through approaches based on hybrid zero dynamics (HZD) and deep reinforcement learning (DRL) [5,6]. While both methods offer substantial strengths, they also present limitations when compared to our proposed MPC approach under similar conditions [7]. HZD provides a systematic framework for ensuring periodic, stable gaits by constraining the system dynamics to a lower-dimensional surface, often resulting in the center of mass (CoM) height being kept relatively constant. This restriction simplifies the control process but limits the method’s flexibility in handling dynamic tasks where the CoM needs to vary, such as jumping or transitioning between different gaits.

DRL, on the other hand, has been used to learn complex, nonlinear behaviors by allowing the robot to interact with its environment and optimize its actions through trial and error. While DRL can adapt to a wide range of tasks, it is computationally expensive, relying heavily on GPU-based parallel computation to handle large-scale iterative training. Training DRL models often requires significant time due to the extensive environmental interactions needed for policy convergence. For example, a recent study by Gu et al. (2023) demonstrated that training a DRL algorithm for bipedal robot locomotion took approximately 6 h to collect 40 million samples in a parallelized simulation environment using 16 workers on an NVIDIA RTX 3090 GPU cluster (manufactured by NVIDIA Corporation, Santa Clara, CA, USA) [8]. Moreover, retraining is typically necessary when reward function parameters are modified, further adding to the computational burden.

In contrast, model predictive control (MPC) leverages CPU-based parallel computation and operates within defined physical constraints, making it particularly efficient for real-time tasks [9]. Unlike DRL, which requires pretraining, MPC performs iterative optimization during runtime, allowing for quick adjustments to control parameters without the need for retraining. This makes MPC highly suitable for tasks involving dynamic changes in CoM height, such as jumping, where rapid feedback and low-latency responses are critical. Each optimization cycle in MPC typically requires only 10–15 milliseconds, ensuring real-time applicability even in dynamically changing conditions. The structured approach of MPC also ensures better handling of tasks with well-defined objectives, such as transitioning between walking and jumping, further demonstrating its adaptability to bipedal robot locomotion challenges.

For these reasons, MPC was chosen as the primary method in this study to address the specific challenges of controlling dynamic motions in bipedal robots.

### 1.3. Objectives and Methodologies

The objective of this study is to develop a bipedal robot controller that generates precise walking and jumping motions using shooting method-based model predictive control (MPC). The approach focuses on optimizing the robot’s ability to perform these motions efficiently and accurately, without requiring extensive redesign or retraining for different tasks.

By employing the shooting method within the MPC framework, the proposed method provides a new, adaptable solution for generating various gaits.

Custom-designed motion sequences are used as input for the controller. The shooting method minimizes the objective function, representing the deviation between the desired and actual motion trajectories. The robot’s state, including joint positions and velocities, is monitored, and the controller outputs real-time control torques to achieve the specified motion. The optimization problem is solved using the Feasibility-Driven Differential Dynamic Programming (FDDP) algorithm, ensuring more accurate control over the robot’s movements and enhancing its ability to perform complex walking and jumping tasks efficiently.

### 1.4. Related Works

#### 1.4.1. Constraint Handling

Xie et al. (2017) proposed the Constrained Differential Dynamic Programming (CDDP) algorithm, which extends the standard DDP to accommodate arbitrary nonlinear constraints on state and control variables [10]. However, CDDP often suffers from computational inefficiency when applied to high-dimensional systems, as the complexity of handling nonlinear constraints increases exponentially with the number of degrees of freedom. This makes it less suitable for real-time applications, especially in highly dynamic environments. Howell et al. (2019) developed ALTRO, a solver for constrained trajectory optimization that enhances the iterative linear quadratic regulator (iLQR) with an augmented Lagrangian approach (AL-iLQR) [11]. While ALTRO manages constraints more effectively than standard methods, it struggles with convergence in scenarios with highly nonlinear dynamics or complex terrain interactions, limiting its applicability to real-world bipedal locomotion tasks.

Kleff et al. (2022) tackled the challenge of handling contact dynamics in arbitrary frames, which is essential for tasks requiring precise force application, such as polishing [12]. They formulated the rigid contact model’s derivatives in user-defined frames and integrated them into the Crocoddyl optimal control solver, effectively managing contact tasks within a model predictive control (MPC) framework. This approach enables contact tasks to be expressed in a frame centered at the contact point, simplifying the task design and ensuring accurate force application. Together, these methods provide a solid foundation for constrained trajectory optimization in bipedal robots, enabling them to perform dynamic tasks in complex environments with improved stability and precision. Building on these insights, our study adopts the Feasibility-Driven Differential Dynamic Programming (FDDP) algorithm within the MPC framework, focusing on real-time feasibility under dynamic conditions, which is particularly important for bipedal locomotion.

#### 1.4.2. MPC Application

MPC is widely used for optimizing control in dynamic environments due to its ability to handle system constraints and uncertainties. It has applications across robotics, autonomous driving, and multi-robot systems.

Meduri et al. (2023) explored the application of nonlinear MPC through their BiConMP framework, designed for whole-body motion planning in humanoid robots. This framework successfully enabled complex tasks such as walking and jumping by optimizing trajectories while addressing dynamic constraints, showcasing MPC’s adaptability in challenging robotic environments [13].

Zhou et al. (2023) extended MPC’s capabilities to autonomous driving by proposing an interaction-aware MPC method. Their approach incorporated multi-modal uncertainties to improve navigation in traffic, a key factor for safe and efficient vehicle operation. Around the same time, Mohseni et al. (2020) introduced a cooperative MPC framework tailored for multi-vehicle coordination, optimizing traffic flow and safety through real-time path adjustments [14,15].

Nair et al. (2022) focused on applying stochastic MPC to address navigation uncertainties at traffic intersections. By integrating multi-modal predictions, their work illustrated MPC’s ability to handle unpredictable scenarios in dynamic environments, further highlighting its potential for scalability in multi-agent systems [16].

These contributions demonstrate the versatility and robustness of MPC as a control strategy across various fields. Its ability to predict system behavior, handle constraints, and adapt to dynamic conditions makes it particularly effective for solving complex control problems, including those encountered in bipedal robot locomotion.

#### 1.4.3. MPC-FDDP Algorithms

The integration of model predictive control (MPC) with Feasibility-Driven Differential Dynamic Programming (FDDP) represents a significant advancement in robotics, particularly for legged robots. This combined approach addresses the challenges of dynamic systems with constraints, offering robust and efficient solutions for trajectory optimization. Tassa et al. (2014) introduced Control-Limited Differential Dynamic Programming (CLDDP), which effectively handles control constraints. However, CLDDP’s performance is sensitive to the quality of the initial trajectory, and poor initial guesses often lead to suboptimal solutions or require many iterations to converge, impacting its real-time viability [17]. Building on this, Carpentier and Mansard (2018) refined DDP by improving the gradient calculations for rigid body dynamics, but this approach can be computationally intensive, especially for high-degree-of-freedom robots such as humanoids. Additionally, rigid body models are limited in their ability to capture the full complexity of bipedal locomotion in dynamic, real-world environments [18].

Dantec et al. (2021) further advanced this field by integrating whole-body model predictive control (WB-MPC) with memory, which uses past motion data for faster and more accurate predictions, which are crucial for real-time dynamic walking in humanoid robots [19]. Mastalli et al. (2022) introduced the BOX-FDDP algorithm, emphasizing feasibility maintenance during optimization to ensure dynamic feasibility under control constraints, making it suitable for real-time applications in legged robots [20]. They demonstrated the use of predictive control for agile maneuvers, highlighting the potential of combining MPC with DDP to handle high-dimensional and dynamic tasks effectively.

Mastalli et al. (2023) advanced these concepts further with an inverse-dynamics MPC approach using nullspace resolution to optimize the control inputs while respecting both physical and operational constraints [21]. This method significantly enhances the robustness of control strategies for legged robots. Additionally, Claraco (2023) provided a comprehensive tutorial on SE(3) transformation parameterizations, which are essential for accurately modeling and controlling robotic systems in complex motion scenarios [22]. Mansard (2023) emphasized a feasibility-prone DDP approach to improve the stability and convergence in DDP algorithms, making them more suitable for real-time MPC applications in robotics [23].

Bergonzani et al. (2023) validated these integrated methods through experimental investigations on the RH5 humanoid robot, demonstrating the practical effectiveness of combining MPC with DDP to achieve fast dynamic walking [24]. These studies collectively enhance the feasibility, stability, and efficiency of control strategies, enabling legged robots to perform complex and dynamic tasks with greater agility and robustness. This body of work sets the stage for future improvements in autonomous robotic systems, promising enhanced performance in a wide range of applications, from industrial automation to search and rescue missions.

These studies highlight the potential of integrating FDDP into MPC frameworks to address control and feasibility challenges. In this study, we further explore this integration by tailoring the MPC-FDDP approach to bipedal locomotion, balancing computational efficiency and robustness to meet the demands of real-time motion planning.

### 1.5. Contributions of This Paper

The main contributions of this paper are as follows:

1. The jumping and walking actions of the studied robot were meticulously broken down and designed employing Bezier curves to smooth and optimize the desired motion trajectories, thus minimizing the robot’s collision with the ground during movement.
2. A specific cost function was designed for the robot’s jumping movements, and in practical experiments, the cost was reduced to a sufficiently low level.
3. An MPC controller framework was designed, integrating MPC with the shooting method, and equipped with an FDDP solver to solve the trajectories.
4. The feasibility of the algorithm was verified in two different simulation environments.

## 2. Methodology

Model predictive control (MPC) offers significant advantages in addressing the gait problems of bipedal robots. MPC is capable of handling multi-objective optimization, incorporating constraints, and predicting future states, making it an ideal approach for generating stable and efficient walking patterns [25]. By continuously updating the control actions based on real-time feedback, MPC ensures a robust performance even in dynamically changing environments. This section will provide a detailed description of the design of the proposed MPC controller, focusing on its architecture, the specific methods employed, and the algorithms used to achieve the desired bipedal locomotion. The controller integrates advanced techniques such as the shooting method and FDDP solver to solve for the optimal trajectories, ensuring both stability and efficiency in the robot’s movements.

### 2.1. Model Predictive Control

The controller in this study is built upon the model predictive control (MPC) framework, an optimal control method designed to achieve the best possible system state while satisfying predefined constraints imposed by the real-world environment. The optimal state is defined according to the specific task requirements; for example, when generating walking or jumping motions for bipedal robots, maintaining balance stability is crucial to prevent falling. The priority of different objectives, such as stability and motion accuracy, can be adjusted by modifying the task weighting parameters within the cost function.

**Figure 2:**
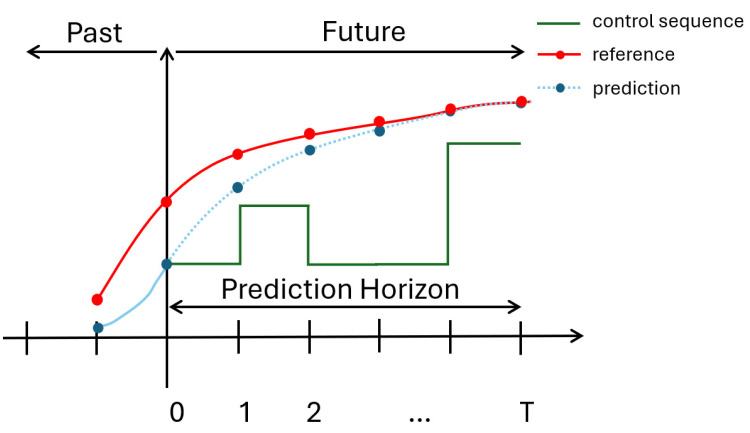
A schematic of model predictive control.

As illustrated in Figure 2, MPC operates through three main steps: prediction, optimization, and application. It uses the dynamic model of the system to predict future behavior over a finite time horizon. Control actions are then determined by solving an optimization problem that minimizes a predefined cost function while adhering to the given constraints.

In the prediction step, the future state of the system is predicted based on its model and control sequence. The system evolves from its discrete-time dynamics:

(1) $$\left.x_{t+1}=f(x_{t},u_{t}\right)$$

where $x_{t}$ represents the current dynamic system state. $u_{t}$ represents the input of control. The next dynamic system state, $x_{t+1}$, is predicted from $u_{t}$ and the dynamic model f.

To evaluate the system state and control input, an instantaneous cost function is defined as follows:

(2) $$c\left(x_{t},u_{t}\right)$$

Which measures the cost at a single time step t. This cost function typically includes terms for tracking errors and control efforts, reflecting the objectives of the control task.

The total cost over a finite time horizon t=0 to t=T is then calculated as follows:

(3) $$J\left(x_{0:T},u_{0:T}\right)=\sum_{t=0}^{T}\left.c(x_{t},u_{t}\right)$$

where $J\left(x_{0:T},u_{0:T}\right)$ represents the cumulative cost function, $x_{0:T}$ is the sequence of system states, and $u_{0:T}$ is the sequence of control inputs over the prediction horizon.

Solving the finite-horizon optimal control problem, which involves optimizing a fixed-length trajectory, is typically referred to as planning or trajectory optimization.

As shown in Figure 2, in the optimization step, the planner optimizes the trajectory based on the optimization objective, obtaining the optimal control sequence $u_{0},u_{1},\ldots ,u_{T}$. Subsequently, in the application step, the first step of the optimal control sequence is applied to the system.

These three steps are repeated during each subsequent time step using the updated system state for new predictions and continuously performing rolling optimization.

### 2.2. Contact-Forward Dynamics Model

After establishing the model predictive control (MPC) framework, the next step involves developing the contact-forward dynamics model, which underpins the predictive tasks in MPC. The initial phase of MPC requires an accurate dynamic model to forecast the system’s behavior; hence, deriving a precise contact-forward dynamics model is essential.

The contact-forward dynamics model captures the motion of a robotic system under various forces and constraints, such as walking or jumping, while interacting with the environment. It includes the effects of contact forces, inertia, Coriolis and centrifugal forces, and gravity [26]. This model enables the prediction of the robot’s future states and the optimization of control inputs.

The derivation begins with the Lagrangian formulation to describe the system’s motion equations, followed by the integration of contact constraints to ensure realistic environmental interactions. The model is then linearized for efficient use in the MPC framework.

Forward dynamics, which determines system motion in response to applied torques, is expressed in a Lagrangian form:

(4) $$\dot{v}=M^{-1}(S^{T}\tau -C(q,\dot{q})\dot{q}-g(q)+J_{c}^{T}\lambda )$$

In this formulation, λ represents the contact force, which is the force exerted by the environment on the robot at the contact points. $J_{c}$ is the Jacobian matrix at the point of external force application, describing the relationship between the contact point and the robot’s joints, and is used to transform contact forces into joint forces. M is the joint space inertia matrix, indicating the inertial properties of the robot in the joint space and reflecting the impact of mass and distribution on the robot’s dynamic behavior. C is the Coriolis force matrix, which accounts for the Coriolis and centrifugal forces generated due to the robot’s motion. g represents the gravitational torque, which is the torque acting on the joints due to gravity. τ is the generalized torque produced by the actuators to drive the robot’s joint movements. $\dot{v}$ is the generalized joint acceleration, representing the rate of change in joint velocities induced by the applied torques. Finally, v and q denote the current generalized joint velocity and generalized joint angle, respectively. This formulation allows for the calculation of the robot’s motion state under given forces and torques, which is crucial for predicting the robot’s behavior under specific control inputs, especially in the design and validation of control strategies.

To reduce computational complexity while maintaining essential physical accuracy, the model can be simplified as follows:

(5) $$\begin{array}{c} \overset{˙}{v}=M^{-1}(\tau_{b}+J_{c}^{T}\lambda )\\ \tau_{b}=S^{T}\tau -C(q,\dot{q})\dot{q}-g(q) \end{array}$$

This is a simplified model because it explicitly solves for the joint accelerations $\dot{\left(v\right)}$ by isolating the inverse of the joint space inertia matrix ${(M}^{-1})$. Instead of recomputing the full dynamic equations at every step, this formulation reduces the computational burden by precomputing terms like the Coriolis matrix $\left(C\right)$ and gravitational torque (g), which are common across iterations. Such precomputation streamlines the process for real-time control, where efficiency is critical.

To ensure that the model accurately reflects physical interactions, contact constraints are introduced. Assuming rigid contacts, where the contact point remains fixed during motion, the contact position function and Jacobian matrix are defined as follows:

(6) ϕ(q)=0

(7) $$J_{c}=\frac{\partial \phi }{\partial q}$$

Under this rigid constraint assumption, the first and second derivatives of the contact position function are zero:

(8) $$\begin{array}{c} J_{c}v=0\\ {\overset{˙}{J}}_{c}v+J_{c}\overset{˙}{v}=0 \end{array}$$

To enhance the numerical integration stability, a PD (Proportional-Derivative) coefficient is introduced, modifying the above constraints to the following:

(9) $$J_{c}\overset{˙}{v}=-a_{c}$$

where $a_{c}$ is the desired acceleration in the constraint space, and according to the conclusion of *J*. Baumgarte, $a_{c}$ can be expressed as follows [27]:

(10) $$a_{c}=a_{\lambda (c)}-\alpha^{c}M_{\lambda (c)}^{ref}\Theta M_{\lambda (c)}-\beta \lambda_{\left(c\right)}$$

After deriving the Lagrangian formulation of the forward dynamics and contact constraints, it is essential to integrate these equations to form a comprehensive dynamic model. This combined equation describes the system’s behavior under forces and constraints, which is essential for accurate predictions and control.

The combined equations merge the dynamics and contact constraints to solve for the system’s acceleration and contact forces. The specific form is as follows:

(11) $$\left[\begin{array}{c} \dot{v}\\ -\lambda \end{array}\right]={\left[\begin{array}{cc} M & J_{c}^{T}\\ J_{c} & 0 \end{array}\right]}^{-1}\left[\begin{array}{c} \tau_{b}\\ -a_{c} \end{array}\right]=\left[\begin{array}{c} y(x,\tau )\\ -g(x,\tau ) \end{array}\right]$$

If $J_{c}$ is full rank, the equation has a unique solution. However, constraints often outnumber the degrees of freedom, leading to multiple solutions. According to the conclusions from C. Mastalli et al. [21], solving the above equation directly yields the following:

(12) $$\begin{array}{c} y(x,\tau )=M^{-1}(\tau_{b}+J_{c}^{T}g(x,\tau ))\\ g(x,\tau )={\hat{M}}^{-1}(a_{c}-J_{c}M^{-1}\tau_{b}) \end{array}$$

In this context, $M^{-1}$ is the operational space inertia matrix, with $\hat{M}=J_{c}M^{-1}J_{c}^{T}$. Using Cholesky decomposition, we can solve for the inverse matrices ${\hat{M}}^{-1}$ and $M^{-1}$, thus obtaining the system’s acceleration and contact forces.

Based on the chain rule and following the guideline, the combined equations derived earlier are linearized. The linearization process involves computing partial derivatives and approximating the system’s behavior around a nominal trajectory. This linearization simplifies the optimization by converting nonlinear dynamics into a quadratic programming (QP) form, reducing computational complexity for real-time applications.

The linearized model is obtained by calculating the partial derivatives of the system’s dynamics with respect to the states and inputs. The equations for linearization are as follows:

(13) $$\begin{array}{c} \delta \overset{˙}{v}=\left[\begin{array}{cc} \frac{\partial \dot{v}}{\partial \tau_{b}} & \frac{\partial \dot{v}}{\partial a_{c}} \end{array}\right]\left[\begin{array}{c} \frac{\partial \tau_{b}}{\partial x_{c}}\\ \frac{\partial a_{c}}{\partial x} \end{array}\right]\delta x+\left[\begin{array}{cc} \frac{\partial \dot{v}}{\partial \tau_{b}} & \frac{\partial \dot{v}}{\partial a_{c}} \end{array}\right]\left[\begin{array}{c} \frac{\partial \tau_{b}}{\partial u_{c}}\\ \frac{\partial a_{c}}{\partial u} \end{array}\right]\delta u\\ \delta \lambda =\left[\begin{array}{cc} \frac{\partial \lambda_{c}}{\partial \tau_{b}} & \frac{\partial \lambda_{c}}{\partial a_{c}} \end{array}\right]\left[\begin{array}{c} \frac{\partial \tau_{b}}{\partial x}\\ \frac{\partial a_{c}}{\partial x} \end{array}\right]\delta x+\left[\begin{array}{cc} \frac{\partial \lambda_{c}}{\partial \tau_{b}} & \frac{\partial \lambda_{c}}{\partial a_{c}} \end{array}\right]\left[\begin{array}{c} \frac{\partial \tau_{b}}{\partial u_{c}}\\ \frac{\partial a_{c}}{\partial u} \end{array}\right]\delta u \end{array}$$

The partial derivatives in the above equations, $\frac{\partial \dot{v}}{\partial \tau_{b}}$, $\frac{\partial \dot{v}}{\partial a_{c}}$, $\frac{\partial \lambda_{c}}{\partial \tau_{b}}$, and $\frac{\partial \lambda_{c}}{\partial a_{c}}$, are obtained through the Recursive Newton–Euler Algorithm (RNEA) and forward dynamics calculations. By manually differentiating the original dynamics equations, we obtain the following:

(14) $$\begin{array}{c} \frac{\partial \dot{v}}{\partial \tau_{b}}=M^{-1}-M^{-1}J_{c}^{T}{\hat{M}}^{-1}J_{c}M^{-1}\\ \frac{\partial \dot{v}}{\partial a_{c}}=M^{-1}J_{c}^{T}{\hat{M}}^{-1}\\ \frac{\partial \lambda }{\partial \tau_{b}}={\hat{M}}^{-1}J_{c}M^{-1}\\ \frac{\partial \lambda }{\partial a_{c}}={\hat{M}}^{-1} \end{array}$$

The linearized model can then be expressed in the following form:

(15) $$\left[\begin{array}{c} \delta \dot{v}\\ -\delta \lambda \end{array}\right]=\left[\begin{array}{c} y_{x}\\ -g_{x} \end{array}\right]\delta x+\left[\begin{array}{c} y_{u}\\ -g_{u} \end{array}\right]\delta u[\begin{array}{c} y_{x}\\ -g_{x} \end{array}]\delta x+[\begin{array}{c} y_{u}\\ -g_{u} \end{array}]\delta u$$

This linearized representation allows for the application of linear control techniques within the MPC framework, facilitating the efficient computation and optimization of the robot’s trajectories under given constraints.

By integrating the dynamic and constraint equations, we can calculate the system’s acceleration and contact forces under the given conditions, providing a complete description of the system’s dynamics. This integration is critical for model predictive control (MPC), as it relies on precise dynamic models for prediction and optimization.

### 2.3. Shooting Method for Trajectory Optimization

**Figure 3:**
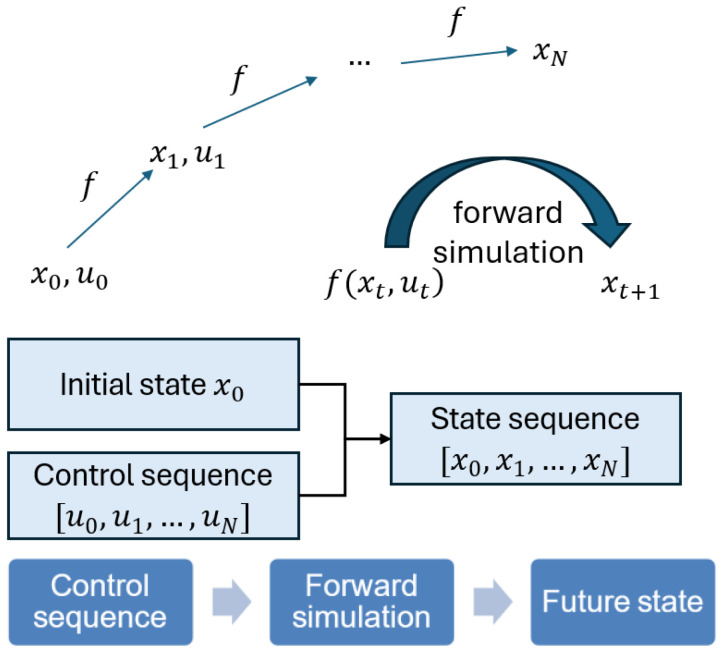
Diagram of shooting method.

The shooting method is a numerical approach for solving finite-horizon optimal control problems in dynamic systems to achieve trajectory optimization [28]. As shown in Figure 3, the shooting method uses the control sequence as the decision variable and enforces dynamics through forward simulation. It simulates a system using the current state and initial control inputs to predict its future state trajectory. Then, based on the predicted trajectory and the predefined cost function, it evaluates the effectiveness of the current control inputs and adjusts them to minimize the cost function. This process is repeated iteratively until the predicted trajectory meets the optimization requirement.

There are two kinds of shooting methods, which means that when applying the shooting method to this problem, we can use either the single shooting method or the multiple shooting method.

The single shooting method involves guessing all the initial control inputs at once and then using numerical integration to calculate the system’s state trajectory [29]. This process continues to adjust the initial guesses until the system’s final state matches the desired boundary conditions.

The single shooting method involves rolling out the system’s dynamics by sequentially applying control inputs, starting from an initial state. This method can be mathematically represented as follows:

(16) $$x_{i+1}=f(f\left(\ldots f\left(f\left(x_{0},u_{0}\right),u_{1}\right)\ldots ,u_{i-1}\right),u_{i})$$

As shown in the equation above, given the initial state $x_{0}$ and a sequence of control inputs U, all the states x are computed through a single roll-out process. If the problem involves T time steps, the states need to be computed repeatedly by integrating over the entire time horizon.

**Figure 4:**
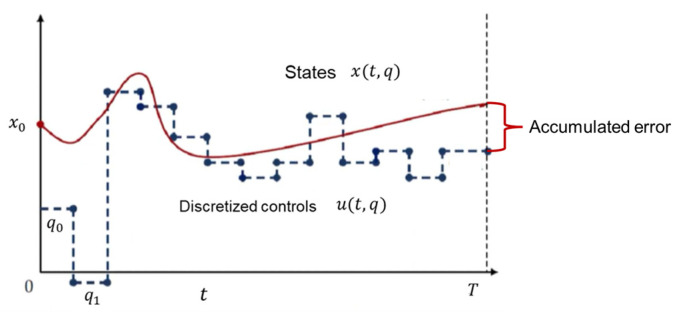
Illustration of single shooting.

However, this approach can lead to a significant issue: the accumulation of errors over long time horizons. With repeated integrations, even small numerical errors can accumulate, potentially leading to significant deviations from the true trajectory and, in some cases, causing divergence. This error accumulation can undermine the accuracy and stability of the solution, especially for complex and high-dimensional systems. For example, in the jumping motions designed in this paper, the required roll-out steps exceed 50 to capture the full dynamics of the motion. Figure 4 shows the process of single shooting.

Considering the disadvantages of the single shooting method, such as the error accumulation over long time horizons and potential divergence, we ultimately chose the multiple shooting method. The multiple shooting method addresses these issues by dividing the time interval into several smaller sub-intervals and optimizing each sub-interval independently. This approach offers several advantages: it improves convergence by breaking down the problem into smaller segments, allowing the optimization process to converge to the global optimum more effectively and reducing the risk of becoming trapped in local optima; it reduces error accumulation, since each sub-interval is independently optimized, minimizing the propagation and accumulation of numerical errors that are common in single shooting; and it increases the robustness by handling complex and high-dimensional systems more effectively, providing better stability and reliability in the optimization process [30].

In the multiple shooting method, each step involves correcting the state trajectory within a sub-interval. The method computes a residual for each sub-interval to ensure continuity and accuracy across the entire time horizon. The residual is defined as follows:

(17) $${\bar{f}}_{k+1}=f(x_{k},u_{k})-x_{k+1}$$

In this formula, ${\bar{f}}_{k+1}$ represents the residual at step k+1, where $f(x_{k},u_{k})$ is the state at the next time step, computed using the current state $x_{k}$, and the control input $x_{k+1}$ is the actual state at the next time step. This residual captures the discrepancy between the predicted state $f(x_{k},u_{k})$ and the actual state, allowing the optimization process to correct the trajectory iteratively.

By minimizing these residuals across all sub-intervals, the multiple shooting method ensures that the overall state trajectory is accurate and stable.

**Figure 5:**
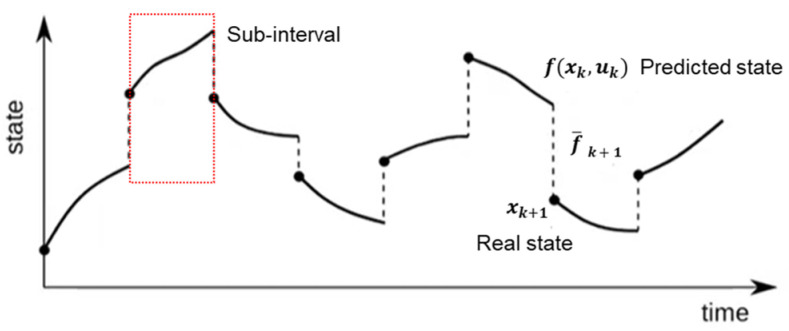
Illustration of multiple shooting.

Figure 5 shows how multiple shooting works and how the residual is defined.

### 2.4. Optimization Objective

In this study, the Crocoddyl framework is used to build the controller [31]. Crocoddyl provides a convenient tool for designing and combining costs to specify the objective function. The form and total cost of these cost functions are defined as follows:

(18) $$J=l_{N}(x_{N})+\sum_{k=0}^{N-1}\int_{t_{k}}^{t_{k}+\Delta t_{k}}l(x,u)dt$$

where $l_{N}(x_{N})$ represents the cost of the final state $x_{N}$, and l(x,u) represents the cost of the state *x* and control input *u* over a time interval.

Three cost functions are used in this study.

#### 2.4.1. CoM and Foot Placement Tracking Cost

The tracking costs are defined as follows:

(19) $$l_{tracking}=w_{CoM}∥CoM(x)-{CoM}_{ref}{∥}^{2}+w_{foot}∥foot(x)-{foot}_{ref}{∥}^{2}$$

where $w_{CoM}$ represents the CoM position under the current robot state x, ${CoM}_{ref}$ represents the reference CoM position in the trajectory, foot(x) represents the foot position under the current state x, and ${foot}_{ref}$ represents the reference foot position in the trajectory.

The center of mass (CoM) tracking cost and foot placement tracking cost aim to minimize the deviation between the robot’s actual movement and the reference trajectory. The CoM tracking cost penalizes errors in the CoM position to ensure that the robot maintains its balance during dynamic movements, such as jumping or sharp turns, and prevents falls due to CoM shifts. The foot placement tracking cost penalizes deviations from the expected landing position, maintaining stability and propulsion efficiency at contact points and preventing unstable gaits or falls.

These cost functions enhance the robot’s stability and control accuracy, allowing it to execute planned movements more precisely and reducing control errors. In dynamic environments or complex terrains, keeping the CoM and foot position close to the reference trajectory helps maintain balance, prevents instability, and improves the overall movement performance.

#### 2.4.2. State and Control Regularization Cost

The regularization costs are defined as follows:

(20) $$l_{\mathrm{regularization}}=w_{x}∥x-x_{\mathrm{ref}}{∥}^{2}+w_{u}∥u-u_{\mathrm{ref}}{∥}^{2}$$

where x represents the current state of the robot, $x_{ref}$ represents the reference state, u represents the control input applied to the robot, and $u_{ref}$ represents the reference control input.

The State and Control Regularization Cost aims to keep the robot’s actions and states close to a desired trajectory, ensuring smooth and stable movement. It minimizes deviations from a reference state and control input, promoting gradual adjustments in the robot’s motion. This helps maintain predictable behavior, which is essential for executing complex tasks or navigating dynamic environments effectively.

By discouraging sudden changes in state or control input, this cost function reduces the risk of instability and unexpected movements. It helps the robot remain efficient and balanced, supporting consistent performance across various conditions while maintaining safety and control during operations.

#### 2.4.3. Impulse Dynamics Cost

The impulse dynamics cost is defined as follows:

(21) $$M\dot{v}+J_{c}^{T}\lambda =Mv^{-}-eJ_{c}v^{-}$$

The impulse dynamics cost involves terms that account for the changes in velocity and forces during contact events, such as impacts or collisions. In this cost function, variables such as $v^{-}$ represent the robot’s velocity immediately before impact, and the velocity immediately after impact is determined by the restitution coefficient e, which reflects the elasticity of the collision. The variable λ denotes the contact impulse, representing the force applied over a short duration during the contact event.

The impulse dynamics cost is designed to manage the robot’s behavior during sudden contact events, such as impacts or collisions, by penalizing deviations from the desired dynamic response. This cost focuses on controlling the changes in the robot’s velocity and forces immediately before and after contact, ensuring that the robot’s movements remain stable and predictable. By doing so, it helps mitigate the disruptive effects of impacts, reducing the risk of instability or loss of balance that could arise from sudden changes in motion.

By optimizing the response to impulses, the cost function supports smoother transitions between different contact states, such as landing after a jump or quickly changing direction. It helps the robot maintain control and efficiency during dynamic tasks, particularly in scenarios where sudden contact with the environment is frequent. This contributes to the robot’s overall agility and robustness, enabling it to handle complex or unexpected interactions more effectively.

#### 2.4.4. Cost Function Weight

To enable the bipedal robot to perform accurate and stable walking and jumping motions, we defined a number of parameters for our designed cost function, as shown in Table 1.

The parameter tuning process was conducted in two simulation environments: Python 3.8 and Gazebo 11. The Python-based simulation used in this study was designed to operate in an open-loop manner, meaning that no feedback from the system’s output was used to adjust the control inputs during execution. This simplifies the tuning process, as larger parameter values can still produce satisfactory results. In contrast, the Gazebo simulation was configured to include feedback mechanisms and account for disturbances, requiring more careful parameter adjustments to maintain stability and achieve the desired performance.

For complex movements, such as jumping, the tuning process was divided into distinct phases. Each phase has different task objectives and thus requires separate parameter adjustments. This ensures that each phase is optimized for its specific goal, whether it is initiating motion, stabilizing mid-air, or ensuring smooth landings. The parameters were fine-tuned for each phase individually to ensure that the robot could perform these transitions smoothly.

Additionally, since the tasks in MPC are interrelated, tuning the parameters in one phase can affect the performance in other phases. Therefore, special attention was given to how adjustments in one phase could impact the overall motion, requiring careful iterative tuning to balance the different objectives across phases.

### 2.5. Feasibility-Driven Differential Dynamic Programming (FDDP)

Because we aimed to minimize the residuals, we chose the Feasible-Driven Differential Dynamic Programming (FDDP) algorithm. FDDP is an optimization algorithm that is particularly suited for control problems in nonlinear dynamic systems [19].

#### 2.5.1. Principle of FDDP

FDDP optimizes control inputs through an iterative process, ensuring that the system meets dynamic constraints while minimizing a given cost function.

Our goal is to achieve the following:

(22) $$x_{k+1}^{*}=f(x_{k}^{*},u_{k}^{*})$$

Thus, we can ensure that the function curve closes and the cost is minimized. To achieve this, we need to adjust the state and control inputs to minimize the residuals. Specifically, we express the corrected part as follows:

(23) $$x_{k+1}+\delta x_{k+1}=f(x_{k}+\delta x_{k},u_{k}+\delta u_{k})$$

Using a Taylor series expansion, we can approximate the function f:

(24) $$f(x_{k}+\delta x_{k},u_{k}+\delta u_{k}){\approx f\left(x_{k},u_{k}\right)+f}_{x_{k}}\delta x_{k}+f_{u_{k}}\delta u_{k}$$

Thus, we have the following:

(25) $$x_{k+1}+\delta x_{k+1}={f\left(x_{k},u_{k}\right)+f}_{x_{k}}\delta x_{k}+f_{u_{k}}\delta u_{k}$$

To ensure that $x_{k+1}^{*}=f(x_{k}^{*},u_{k}^{*})$ holds, we need to establish the following:

(26) $$\delta x_{k+1}=f_{x_{k}}\delta x_{k}+f_{u_{k}}\delta u_{k}+{\bar{f}}_{k+1}$$

Ultimately, we aim to minimize the following objective function:

(27) $$\underset{\mathrm{\delta x},\mathrm{\delta u}}{\min}L=l_{N}\left(\delta x_{N}\right)+\sum_{i=0}^{N-1}l_{i}\left(\delta x_{i},\delta u_{i}\right)\;\delta x_{0}={\bar{x}}_{0}$$

where $l_{N}$ is the terminal cost function and $l_{i}$ is the stage cost function at each time step. The initial conditions and state transition equations are as follows:

(28) $$\delta x_{k+1}=f_{x_{k}}\delta x_{k}+f_{u_{k}}\delta u_{k}+{\bar{f}}_{k+1}$$

In this way, FDDP transforms the original optimization problem into one of correcting the deviations. By iteratively solving for the corrections $\delta x_{0}$ and δu, we can achieve the minimal cost for the actual movement.

#### 2.5.2. Computational Complexity and Real-Time Applicability

The proposed MPC-FDDP framework, implemented using the Crocoddyl library, benefits significantly from the support of parallel computing, which helps accelerate the computational process. The experiments were conducted on a computer with an Intel Core i7-12700KF processor (manufactured by Intel Corporation, Santa Clara, CA, USA and sourced from Amazon Tokyo, Japan) and 32 GB of memory, allowing the use of multi-threaded computation to distribute optimization tasks across multiple cores. This parallel computing setup improved the computational speed of MPC, reducing the typical computation time for each cycle to approximately 10 to 15 ms.

This level of performance suggests that the framework may be able to run in real time on physical robots, provided that sufficient computational resources are available. For example, on platforms with multi-core processors, enabling parallel computing allows optimization tasks to be distributed across multiple threads, reducing the time required for trajectory optimization. Therefore, the MPC-FDDP approach might be suitable for real-time applications in complex dynamic tasks, such as bipedal walking and jumping.

However, the real-time applicability of this method may vary depending on the hardware platform used. On lower-performance embedded systems, such as those commonly found in mobile robots, further optimization techniques, such as code parallelization and using more efficient solvers, may be necessary to maintain the same computational speed. The adaptability of the method to different hardware configurations makes it a flexible option for real-world robotic applications.

## 3. Experiment

### 3.1. Environment Configuration

The primary software packages used include the following:

Crocoddyl 2.0.2: A framework for control optimization in robotics, particularly useful for implementing model predictive control (MPC) and Feasible Differential Dynamic Programming (FDDP).

Pinocchio 3.0.0: A library for the efficient modeling and control of articulated mechanisms, providing tools for kinematics, dynamics, and control tasks.

ROS Noetic Ninjemys: An open-source framework for robot software development, providing libraries and tools for creating robot applications.

Gazebo: A simulation tool that provides a robust environment to test and validate control algorithms.

These tools were crucial for developing, testing, and validating the control algorithms and simulations, ensuring that experiments were conducted efficiently and accurately.

### 3.2. Robot Model

The bipedal robot model employed in this study, named Hector, is an open-source robot model specifically designed to facilitate research in robotic locomotion [32]. Hector is a bipedal robot with 10 degrees of freedom (DoFs), featuring five actuated joints per leg. These joints include abduction and hip joints for 3D rotation and ankle joints for double-leg support, configurations that are similar to those found in the existing literature on advanced bipedal robots. The model’s design allows for a wide range of motions, closely mimicking the flexibility and mobility of human legs.

**Figure 6:**
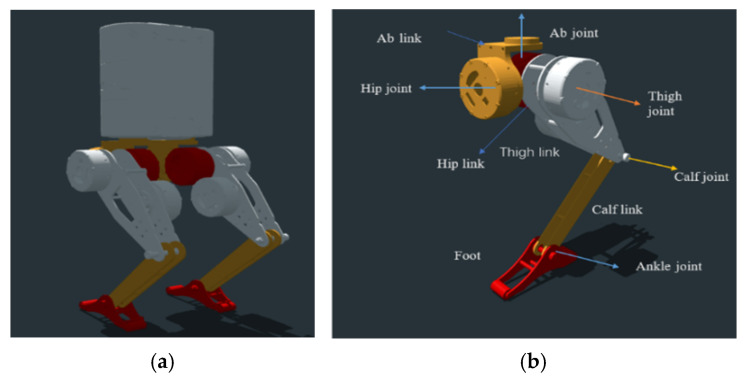
Bipedal robot configuration: (**a**) robot URDF model; (**b**) link and joint configuration of the bipedal robot’s left leg.

The model of the Hector robot and the robot’s leg are shown in Figure 6, while Table 2 presents a detailed overview of the robot’s physical parameters, and Table 3 demonstrates the robot joint configurations.

### 3.3. Experimental Design

To develop human-like locomotion capabilities for the humanoid robot, we designed experiments to generate walking and jumping motions. The primary objective was to create these motions using the MPC controller proposed in this study.

The walking and jumping motions were designed to emulate natural human movements. The proposed MPC controller was applied to generate the required control inputs, allowing the robot to execute these motions accurately and efficiently. The controller dynamically optimized the robot’s trajectories in real time, ensuring smooth transitions and stable behavior throughout the movement sequences.

These experiments aimed to validate the effectiveness of the proposed MPC controller in generating human-like walking and jumping motions, demonstrating its ability to handle complex tasks and adapt to various conditions while maintaining the desired performance.

#### 3.3.1. Walking Motion Design

To generate a natural walking motion for the humanoid robot, we first analyzed the typical human walking process, where the trailing leg moves forward in a repetitive cycle. Similar research has been conducted on human gait analysis and robotic locomotion, which provides valuable insights into the kinematics and dynamics of walking. For instance, Winter’s foundational work on human biomechanics offers detailed characterizations of joint trajectories during walking [33]. Building on these findings, we designed a step cycle that closely mimics human-like walking patterns. Transitioning from a stationary state to walking involves moving one leg forward from a parallel stance, resulting in an initial step length that is half of a full stride. Similarly, when transitioning from walking back to a stationary position, the final step length is also half of a full stride. This requires special consideration for both the initial and final steps, where the displacement and duration are reduced to half of those of a regular stride while maintaining a consistent step height to ensure a natural gait.

To achieve smooth motion trajectories and minimize the risk of collisions, Bézier curves were employed to refine the trajectory design. This mathematical approach allows for the creation of fluid and continuous paths, enhancing the naturalness of the robot’s gait and reducing abrupt transitions that could destabilize the robot.

Based on the robot model used in this study and extensive experimental trials, the following parameters were established to optimize the walking motion: a stride length of 0.1 m, a step height of 0.05 m, a double support phase duration of 0.07 s, and a single support phase duration of 0.31 s. These parameters ensure a balanced and efficient walking pattern.

**Figure 7:**
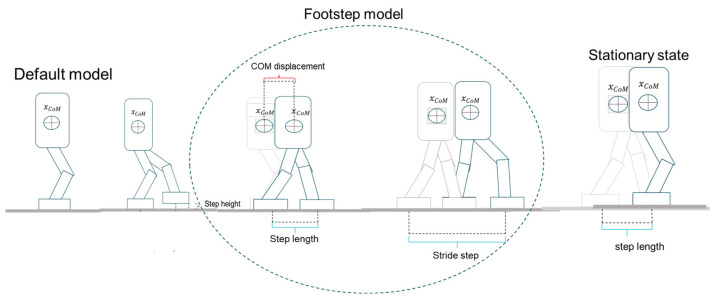
Walking model.

Special attention was given to the transitions between stationary and walking states. The leg and center of mass (CoM) displacements, as well as the time required for these transitions, were halved compared with a normal stride while maintaining a consistent step height. This approach preserves the natural characteristics of the walking gait. The entire walking process is illustrated in Figure 7.

#### 3.3.2. Jumping Motion Design

The jumping motion is divided into four distinct phases: takeoff, ascent, descent, and landing. Each phase addresses specific aspects of the motion to ensure a stable and controlled jump.

Takeoff: During this initial phase, the robot must generate sufficient kinetic energy to leave the ground. This requires coordinated movements of multiple joints to apply a powerful force against the ground, propelling the robot upwards. Proper posture adjustment is crucial to maximize the efficiency of the takeoff and achieve the desired jump height.

Ascent: Once airborne, the robot has limited control, since its feet are no longer in contact with the ground. The primary focus during this phase is to adjust the foot angles to prepare for a stable landing. By positioning the feet at an optimal angle, the robot minimizes the risk of instability upon impact. At the peak of the jump, the center of mass displacement should be half the total jump length, and the foot height should match the pre-set jump height. This design ensures a smooth trajectory and reduces the likelihood of collisions.

Descent: As the robot begins to descend, it continues to adjust the foot angles to ensure that they are parallel to the ground. This preparation is essential for distributing impact forces evenly upon landing, thus reducing the risk of tipping or instability.

Landing: In the final phase, the robot must swiftly adjust its center of gravity to maintain a stable posture upon contact with the ground. Rapid and precise corrections are necessary to prevent tipping or the loss of balance, ensuring a safe and controlled conclusion to the jump.

**Figure 8:**
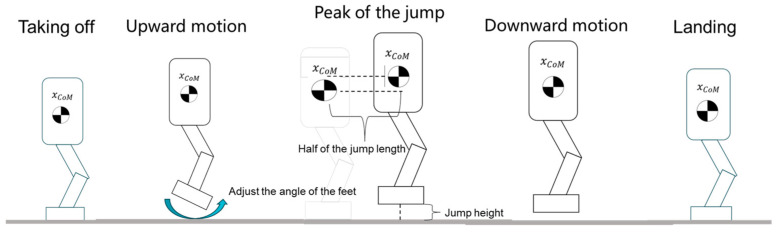
Jumping motion.

By systematically addressing the requirements of each phase, the robot achieves a smooth and stable jumping motion, minimizing the risk of instability and enhancing landing safety. The entire process is depicted in Figure 8.

### 3.4. Experimental Results

This section presents the comprehensive simulation results of the walking and jumping motions performed using both the Python and Gazebo environments. These simulations were essential in validating the effectiveness of the proposed model predictive control (MPC) controller for the bipedal robot model, Hector. By utilizing two distinct simulation platforms, we were able to thoroughly evaluate the robot’s performance in executing these motions under different conditions.

The use of both platforms allowed for an in-depth analysis of the robot’s behavior, confirming the effectiveness of the proposed MPC controller in achieving stable and controlled walking and jumping motions.

#### 3.4.1. Walking

**Figure 9:**
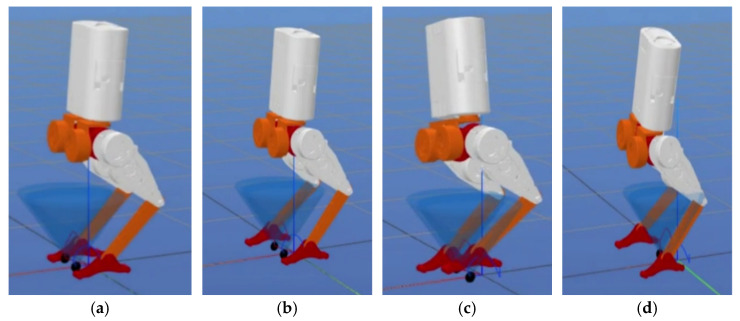
Python environment: (**a**) the robot in a stationary standing position; (**b**) the robot taking a step with its right foot as the first step; (**c**) the robot taking a step with its left foot as the second step; (**d**) the robot taking a step with its right foot as the third step.

**Figure 10:**
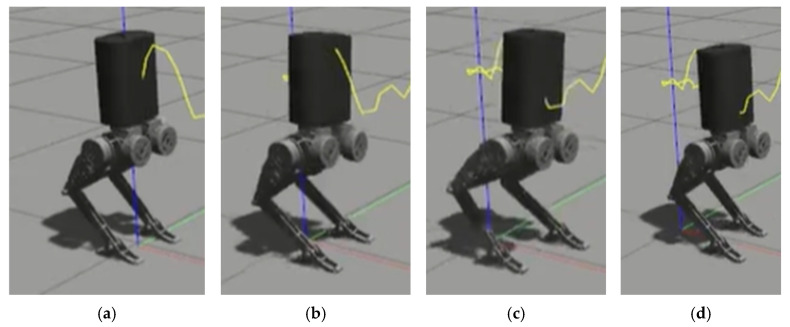
Gazebo environment: (**a**) the robot in a stationary standing position; (**b**) the robot taking a step with its right foot as the first step; (**c**) the robot taking a step with its left foot as the second step; (**d**) the robot taking a step with its right foot as the third step.

Table 4 shows the walking parameters, Figure 9 shows the walking result in Python, and Figure 10 shows the walking result in Gazebo.

In Figure 9, the red line typically represents the X-axis in the world coordinate system, the green line represents the Y-axis, and the blue line represents the Z-axis (vertical direction).

In Figure 10, the red line represents the X-axis, the green line represents the Y-axis, the blue line represents the Z-axis, and the yellow line indicates the trajectory of the robot’s center of mass.

**Figure 11:**
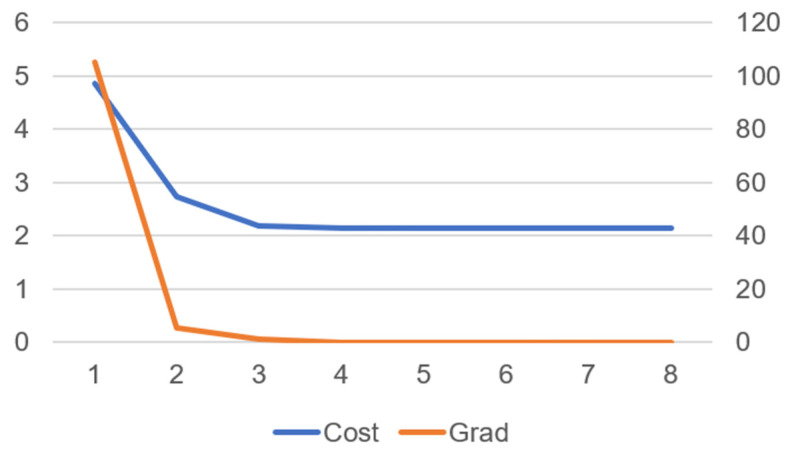
Cost and gradient convergence over iterations in robot walking motion.

Figure 11 shows that as the number of iterations increases, both the cost and gradient gradually decrease, indicating that the optimization process is progressing toward the optimal solution.

The experiments in Python and Gazebo confirmed that the robot could successfully perform walking motions with the parameters set for stride step length, step height, double support phase duration, and single support phase duration.

#### 3.4.2. Jumping

**Figure 12:**
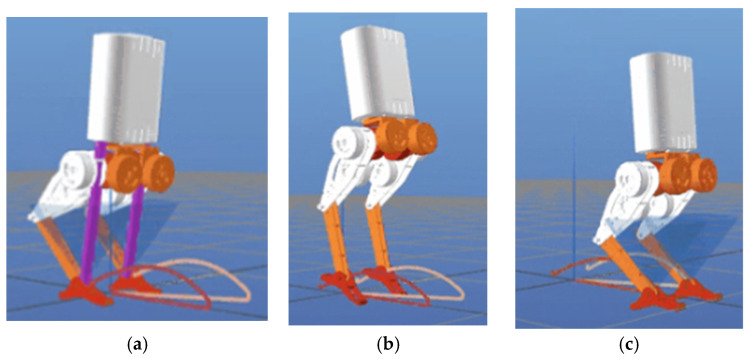
Python environment: (**a**) robot in stationary phase; (**b**) robot in jumping phase; (**c**) robot in landing phase.

**Figure 13:**
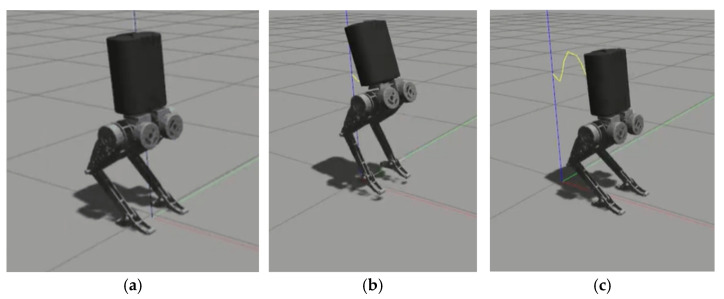
Gazebo environment: (**a**) robot in stationary phase; (**b**) robot in jumping phase; (**c**) robot in landing phase.

Table 5 shows the jumping parameters, Figure 12 shows the jumping result in Python, and Figure 13 shows the jumping result in Gazebo.

In Figure 12, the purple line represents the direction of the contact force during the jumping motion, illustrating the interaction between the robot’s feet and the ground. The red line and yellow line represent the motion trajectories of the robot’s feet, corresponding to their paths during the takeoff, flight, and landing phases.

In Figure 13, Figure 15, and Figure 16, similar to Figure 10, the red line represents the X-axis in the world coordinate system, the green line represents the Y-axis, the blue line represents the Z-axis, and the yellow line indicates the predicted trajectory of the robot’s center of mass.

**Figure 14:**
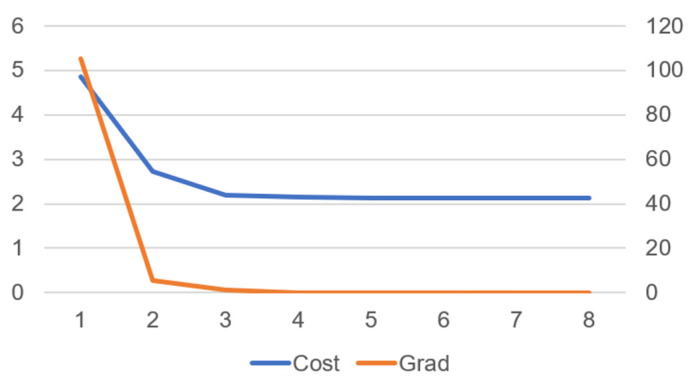
The relationship between the number of iterations and cost and gradient in the jumping motion of the robot.

From Figure 14, it can be observed that as the number of iterations increases, both the cost and gradient gradually decrease, indicating that the optimization process is progressing towards the optimal solution.

### 3.5. Simulation on Low-Friction Surface

To further validate the robustness of the proposed model predictive control (MPC) method, we conducted experiments in a low-friction environment. In real-world scenarios, robots often need to walk or jump on slippery surfaces, so it is essential to test the controller’s performance under such conditions.

#### 3.5.1. Experimental Setup

According to The Physics Factbook, the friction coefficient of a wet, slippery surface ranges from 0.45 to 0.75 [34]. Based on this, we set the ground friction coefficient in the simulation to 0.65 to simulate moderately slippery conditions. The other experimental parameters remained consistent with those in Section 3.4 to ensure comparability.

#### 3.5.2. Experimental Results on Continuous Jumping

**Figure 15:**
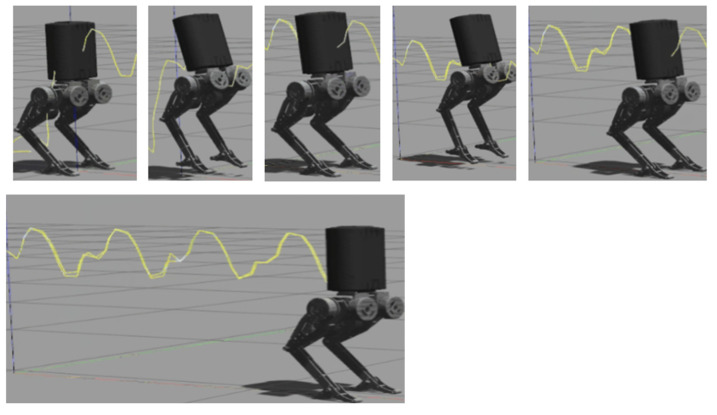
Simulation of continuous jumping on a low-friction surface.

In the low-friction environment, the robot successfully performed continuous jumping simulations. The robot was able to execute multiple jumps, recover stability after each jump, and proceed to the next jump without any issues. Figure 15 illustrates this process, showcasing the robot’s ability to maintain stability and execute smooth transitions between consecutive jumps on a slippery surface.

**Figure 16:**
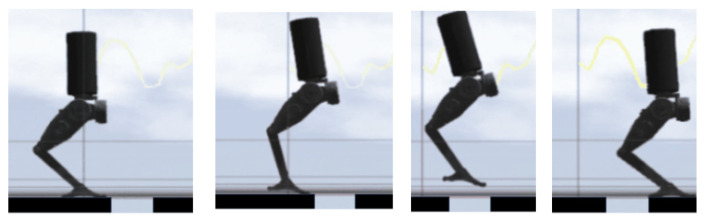
Jumping over a gap of 45 cm.

And Figure 16 shows that the bipedal robot successfully jumps from plane A to plane B, with a jump length of 45 cm.

## 4. Discussion

The results of this study confirm the effectiveness of the proposed model predictive control (MPC) controller in generating human-like walking and jumping motions for the humanoid robot, Hector. The simulations conducted in both the Python and Gazebo environments demonstrated the controller’s ability to achieve stable and controlled movements, aligning with the goal of enhancing humanoid robot locomotion by emulating human gait dynamics.

Our findings build on previous studies in bipedal robot control, such as hybrid zero dynamics (HZD) and deep reinforcement learning (DRL). While HZD requires maintaining a constant center of mass height and DRL demands extensive training data, the proposed MPC controller offers a more adaptable solution, handling complex dynamic tasks such as walking and jumping without the need for frequent retraining or specific initial conditions. This study employed an MPC framework based on the shooting method combined with the Feasibility-Driven Differential Dynamic Programming (FDDP) algorithm. This approach significantly reduced the total cost from the initial values, closely aligning with the desired trajectories and demonstrating the controller’s ability to generate flexible and precise motions in complex environments.

### 4.1. Scalability

The proposed MPC-FDDP framework provides a flexible and adaptable solution for generating bipedal walking and jumping motions. However, the framework’s scalability to more complex motions, such as running and obstacle negotiation, is an important topic that deserves further exploration. This section discusses how the current approach could be extended to accommodate these more advanced tasks and outlines potential future directions for this research.

#### 4.1.1. Scalability to Running Motion

Running presents a set of unique challenges compared with walking or jumping due to the increased dynamic complexity. In running, the robot experiences longer flight phases, higher velocities, and more significant impact forces upon landing. To extend the current MPC-FDDP framework to running, several adjustments would need to be made:

Cost Function Modification: The current cost functions focus on minimizing deviations from the desired trajectory during walking and jumping. For running, the cost function would need to prioritize speed while maintaining balance and stability. The impact of higher velocities on joint torques and foot placements must be accounted for, ensuring that the robot maintains a proper posture during the faster, more dynamic movement.

Flight Phase Handling: Running introduces longer flight phases between foot contacts with the ground. This necessitates enhanced modeling of the robot’s dynamics during the airborne phase and more accurate prediction of the ground contact timing. The MPC framework would need to optimize the control inputs to ensure smooth landings and effective propulsion during takeoff.

#### 4.1.2. Scalability to Obstacle Negotiation

Another potential extension of the current framework is adapting it for more complex tasks such as obstacle negotiation, where the robot needs to dynamically adjust its gait to avoid or step over obstacles. The following modifications would be necessary:

Environmental Perception and Obstacle Detection: To handle obstacles, the robot must first be able to perceive and identify them in real time. Integrating perception systems (e.g., vision sensors or LiDAR) with the MPC-FDDP framework would allow the robot to gather information about its surroundings and dynamically plan its trajectory to avoid obstacles.

Real-Time Path Planning: Once obstacles are detected, the MPC must be capable of real-time path planning. This requires adjusting the robot’s trajectory based on both the obstacle’s location and the robot’s current state. Path planning algorithms such as Rapidly exploring Random Trees (RRTs) or A* could be integrated into the MPC framework to calculate feasible paths that avoid collisions while maintaining stability.

Dynamic Foot Placement Adjustment: The ability to step over or around obstacles depends on accurately adjusting foot placements in real time. The MPC framework would need to include additional constraints to ensure that foot placements are dynamically adjusted based on the size and location of obstacles, without compromising the robot’s balance.

### 4.2. Future Work

While the MPC controller has successfully generated the desired motions in simulated environments, several challenges remain for real-world applications. Issues such as unmodeled dynamics, sensor noise, and hardware limitations could affect the performance in practice. Future work will focus on validating these results through experiments on physical humanoid robots to assess the controller’s real-world performance. This will help refine the MPC framework to handle the inherent uncertainties and complexities in physical systems, such as hardware limitations and environmental disturbances, better.

Additionally, integrating MPC with machine learning techniques could enhance the controller’s adaptability and improve its generalization across diverse tasks and environments. By combining these approaches, we aim to develop a more flexible and robust control system that is capable of operating in real time on a physical robot.

More broadly, this study advances humanoid robot locomotion by developing versatile control strategies that enable robots to handle complex tasks with greater autonomy and efficiency. Future research could expand on these findings by exploring dynamic tasks such as running or climbing and applying the proposed methods to different robot models or hardware platforms. This would further evaluate the scalability and robustness of the shooting method-based MPC framework in achieving human-like mobility across a variety of real-world scenarios.

## Figures and Tables

**Table 1:** Parameters of the cost functions.

Parameter	Jump Stand Mode	Jump Flight Mode	Walk Mode	Walk Double Mode
State weight	1.0	1.0	1.0	1.0
Joint position weight	2.0	2.0	0.1	1.0
Pose weight	1.0	0.015	0.5	2.1
Velocity weight	0.02	0.0	0.01	0.001
Impulse velocity weight	0.01	0.15	0.06	0.06
Impulse joint weight	0.1	0.15	0.005	0.005
State error weight	0.2	0.13	0.13	0.013
Control input weight	0.003	5.0	0.002	0.001
Contact force weight	0.2	2.0	40.0	15.0
Inequality constraint weight	1000.0	1000.0	1000.0	1000.0
Center of mass constraint weight	0.001	0.001	0.01	0.01
Center of pressure control weight	3.0	15.0	120.0	200.0
Impulse force weight	320.0	100.0	60.0	60.0

**Table 2:** Physical parameters of robot.

Parameter	Symbol	Value	Units
Mass	m	16.0	kg
Body Inertia	$I_{xx}$	0.541	$kg\cdot m^{2}$
	$I_{yy}$	0.520	$kg\cdot m^{2}$
	$I_{zz}$	0.069	$kg\cdot m^{2}$
Body Length	$l_{b}$	0.114	m
Body Width	$w_{b}$	0.194	m
Body Height	$h_{b}$	0.247	m
Thigh and Calf Lengths	$l_{1},l_{2}$	0.2	m
Foot Length	$l_{toe}$	0.09	m
	$l_{heel}$	0.05	m
Arm Length	$l_{arm}$	0.20	m
Cane Length	$l_{cane}$	0.52	m

**Table 3:** Joint configurations of robot.

Parameter	Value
Max Joint Speed	21 Rad/s
Max Torque	33.5 N·m
The initial angle of the Shoulder joint around the x-axis	0 deg
Lower Limit	−15 deg
Upper Limit	90 deg
The initial angle of the Shoulder joint around the y-axis	0 deg
Lower Limit	−90 deg
Upper Limit	90 deg
The initial angle of the Elbow joint around the y-axis	−90 deg
Lower Limit	−150 deg
Upper Limit	−10 deg

**Table 4:** Walking parameters of robot.

Walking Parameters	
Time step	0.01 s
Step length	0.12 m
Step height	0.07 m
Time steps for step	33
Time steps for double support	8

**Table 5:** Jumping parameters of robot.

Jumping Parameters	
Time step	0.01 s
Jump length	0.45 m
Jump height	0.25 m
Time steps for ground	35
Time steps for flight	18

## Data Availability

No new data were created or analyzed in this study.
